# Supplementary material for: A Humanized Yeast Phenomic Model of Deoxycytidine Kinase to Predict Genetic Buffering of Nucleoside Analog Cytotoxicity
Source: Genes (Basel). 2019 Sep 30;10(10):770. doi: 10.3390/genes10100770 (PMC6826991; doi:10.3390/genes10100770)
Supplement: Supplementary file 1 [file genes-10-00770-s001.zip › Additional_Files/Additional_File7_GOTermHeatmaps.zip!/Additional_File7_GOTermHeatmaps/ribonucleoprotein_complex_assembly.pdf]

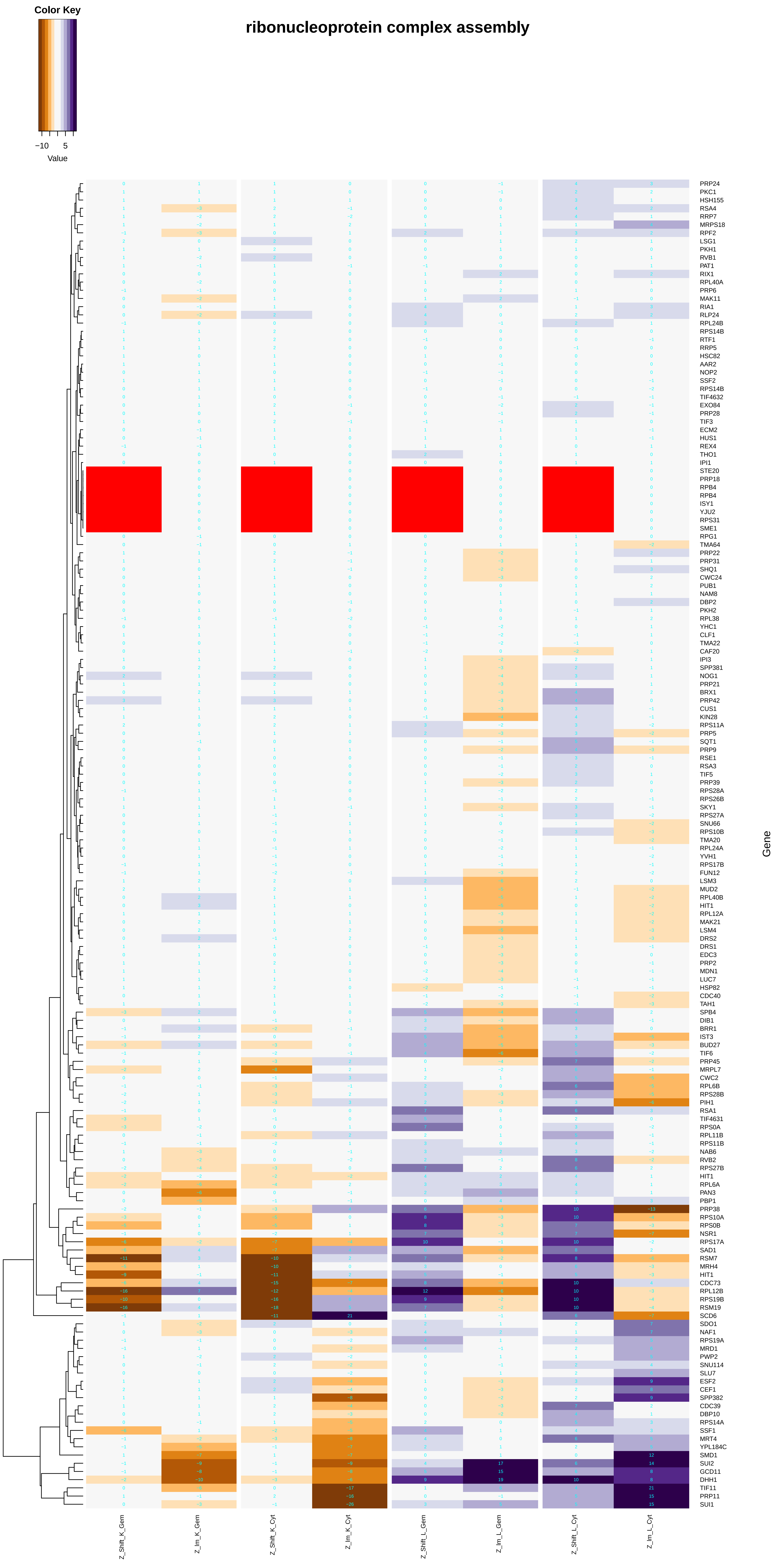

Color Key
−10
5
Value
ribonucleoprotein complex assembly
Gene
Z_Shift_K_Gem
Z_Im_K_Gem
Z_Shift_K_Cyt
Z_Im_K_Cyt
Z_Shift_L_Gem
Z_Im_L_Gem
Z_Shift_L_Cyt
Z_Im_L_Cyt
PRP24
PKC1
HSH155
RSA4
RRP7
MRPS18
RPF2
LSG1
PKH1
RVB1
PAT1
RIX1
RPL40A
RRP6
MAK11
RIA1
RLP24
RPL24B
RPS14B
RTF1
RRP5
HSC82
AAR2
NOP2
SSF2
RPS14B
TIF4632
EXO84
PRP28
TIF3
ECM2
HUS1
REX4
THO1
IPI1
STE20
PRP18
RPB4
RPB4
ISY1
YJU2
RPS31
SME1
RPG1
TMA64
PRP22
PRP31
SHQ1
CWC24
PUB1
NAM8
DBP2
PKH2
RPL38
YHC1
CLF1
TMA22
CAF20
IPI3
SPP381
NOG1
PRP21
BRX1
PRP42
CUS1
KIN28
RPS11A
PRP5
SQT1
PRP9
RSE1
RSA3
TIF5
PRP39
RPS28A
RPS26B
SKY1
RPS27A
SNU66
RPS10B
TMA20
RPL24A
YVH1
RPS17B
FUN12
LSM3
MUD2
RPL40B
HIT1
RPL12A
MAK21
LSM4
DRS2
DRS1
EDC3
PRP2
MDN1
LUC7
HSP82
CDC40
TAH1
SPB4
DIB1
BRR1
IST3
BUD27
TIF6
PRP45
MRPL7
CWC2
RPL6B
RPS28B
PIH1
RSA1
TIF4631
RPS0A
RPL11B
RPS11B
NAB6
RVB2
RPS27B
HIT1
RPL6A
PAN3
PBP1
PRP38
RPS10A
RPS0B
NSR1
RPS17A
SAD1
RSM7
MRH4
HIT1
CDC73
RPL12B
RPS19B
RSM19
SCD6
SDO1
NAF1
RPS19A
MRD1
PWP2
SNU114
SLU7
ESF2
CEF1
SPP382
CDC39
DBP10
RPS14A
SSF1
MRT4
YPL184C
SMD1
SUI2
GCD11
DHH1
TIF11
PRP11
SUI1

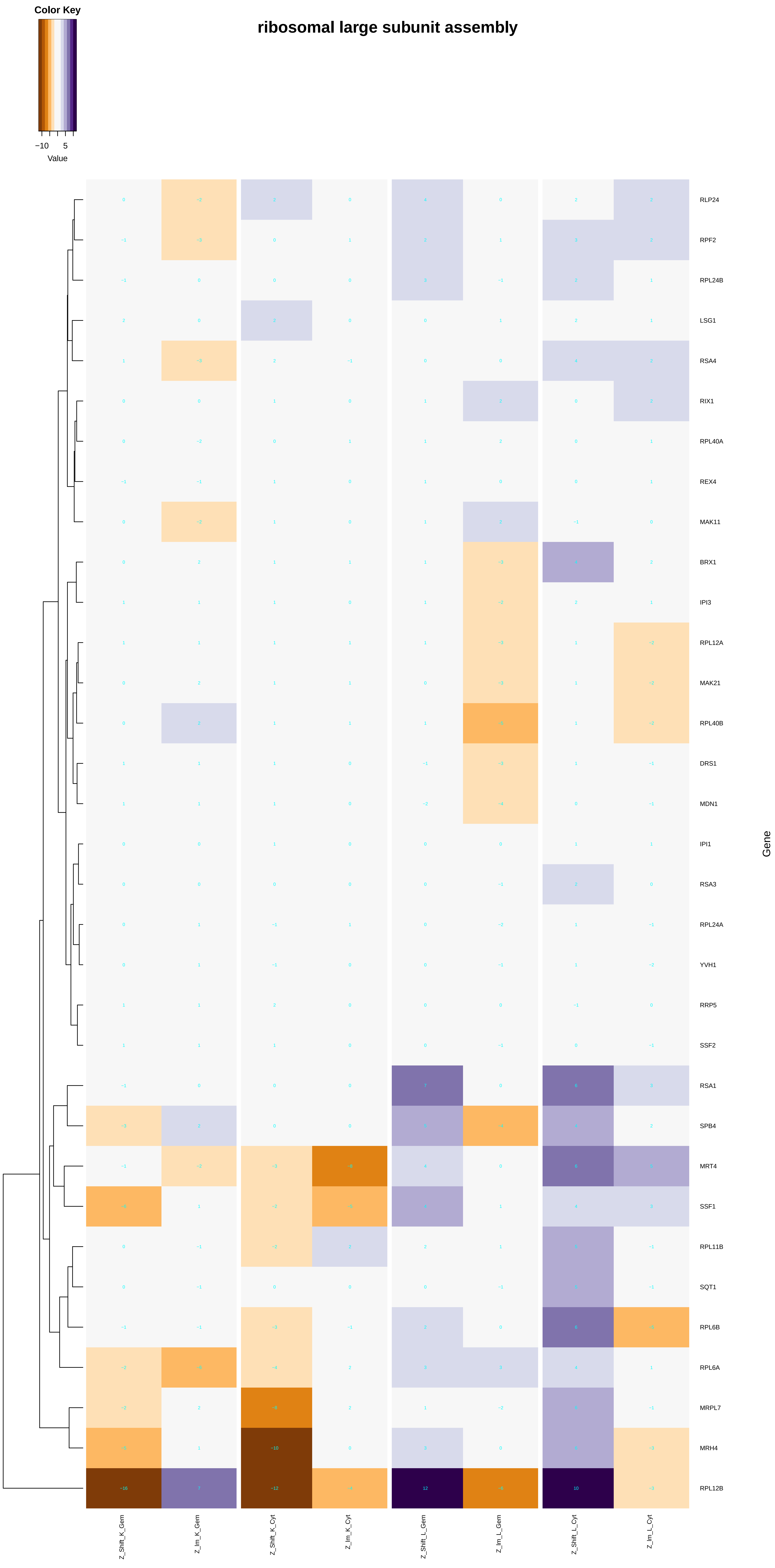

Color Key
−10 5
Value
ribosomal large subunit assembly
RLP24
RPF2
RPL24B
LSG1
RSA4
RIX1
RPL40A
REX4
MAK11
BRX1
IPI3
RPL12A
MAK21
RPL40B
DRS1
MDN1
IPI1
RSA3
RPL24A
YVH1
RRP5
SSF2
RSA1
SPB4
MRT4
SSF1
RPL11B
SQT1
RPL6B
RPL6A
MRPL7
MRH4
RPL12B
Gene
Z_Shift_K_Gem
Z_Im_K_Gem
Z_Shift_K_Cyt
Z_Im_K_Cyt
Z_Shift_L_Gem
Z_Im_L_Gem
Z_Shift_L_Cyt
Z_Im_L_Cyt

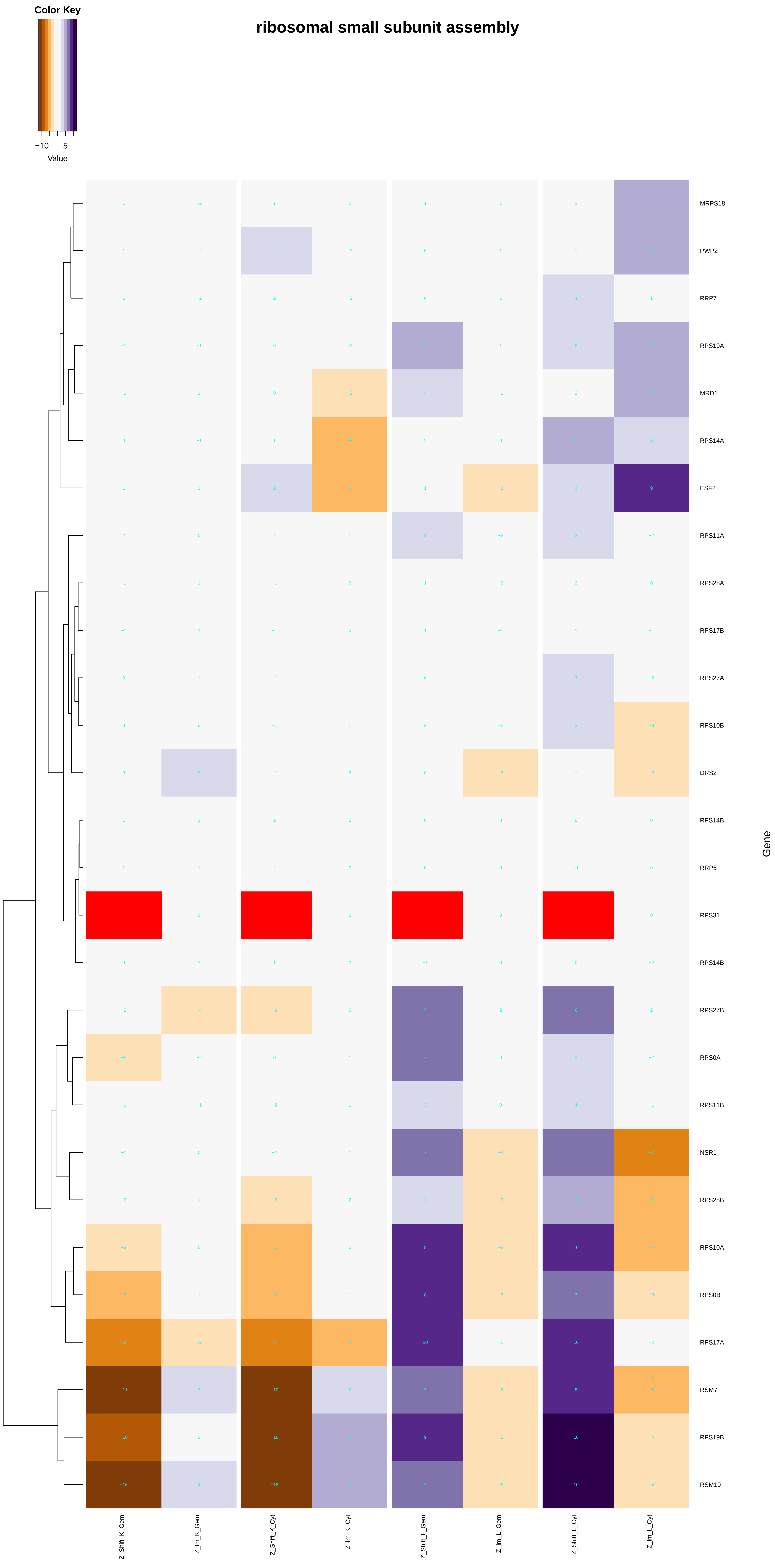
ribosomal small subunit assembly
Color Key
−10
5
Value
MRPS18
PWP2
RRP7
RPS19A
MRD1
RPS14A
ESF2
RPS11A
RPS28A
RPS17B
RPS27A
RPS10B
DRS2
RPS14B
RRP5
RPS31
RPS14B
RPS27B
RPS0A
RPS11B
NSR1
RPS28B
RPS10A
RPS0B
RPS17A
RSM7
RPS19B
RSM19
Gene
Z_Shift_K_Gem
Z_Im_K_Gem
Z_Shift_K_Cyt
Z_Im_K_Cyt
Z_Shift_L_Gem
Z_Im_L_Gem
Z_Shift_L_Cyt
Z_Im_L_Cyt

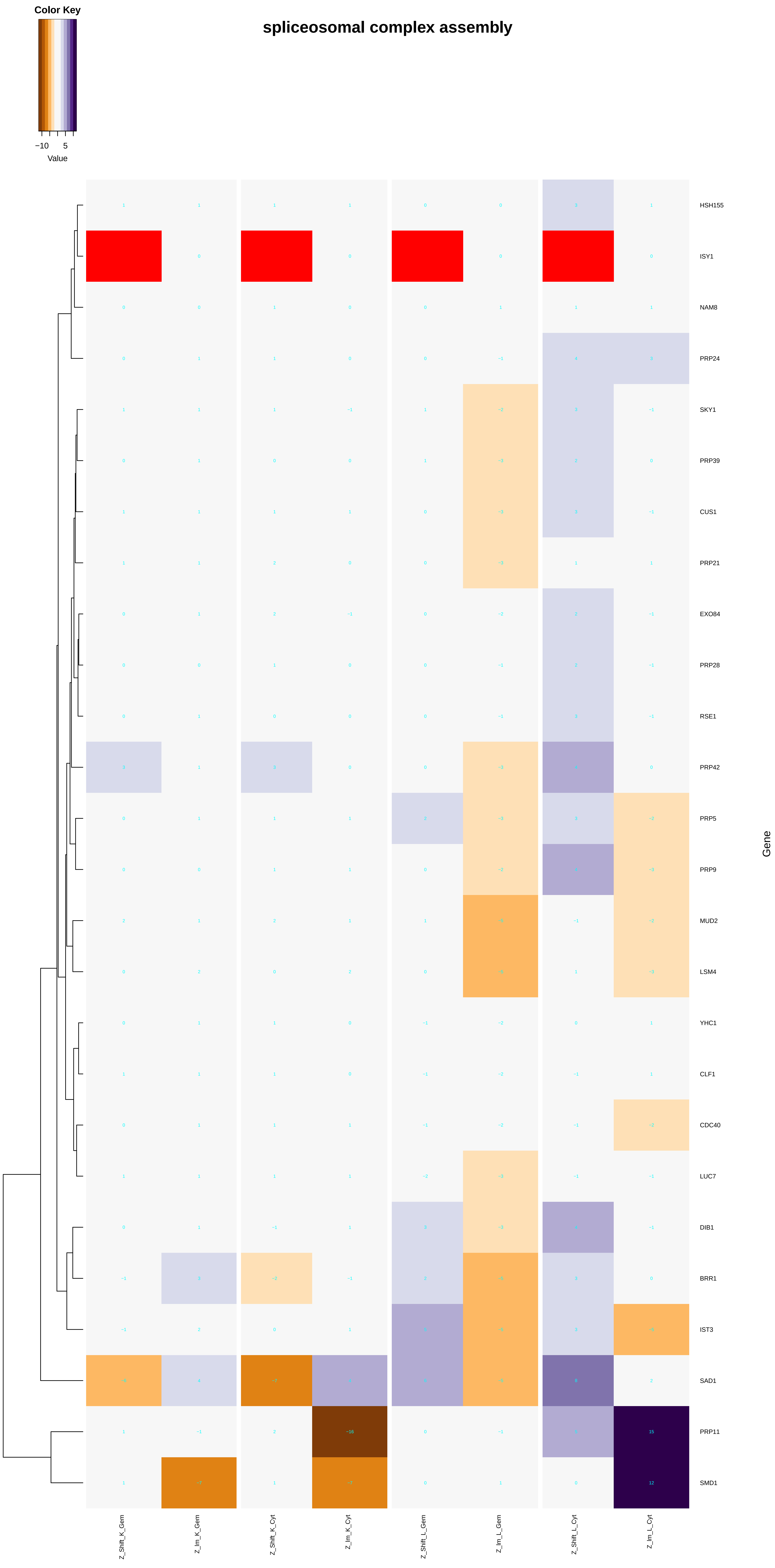
Color Key
−10
5
Value
spliceosomal complex assembly
HSH155
ISY1
NAM8
PRP24
SKY1
PRP39
CUS1
PRP21
EXO84
PRP28
RSE1
PRP42
PRP5
PRP9
MUD2
LSM4
YHC1
CLF1
CDC40
LUC7
DIB1
BRR1
IST3
SAD1
PRP11
SMD1
Gene
Z_Shift_K_Gem
Z_Im_K_Gem
Z_Shift_K_Cyt
Z_Im_K_Cyt
Z_Shift_L_Gem
Z_Im_L_Gem
Z_Shift_L_Cyt
Z_Im_L_Cyt

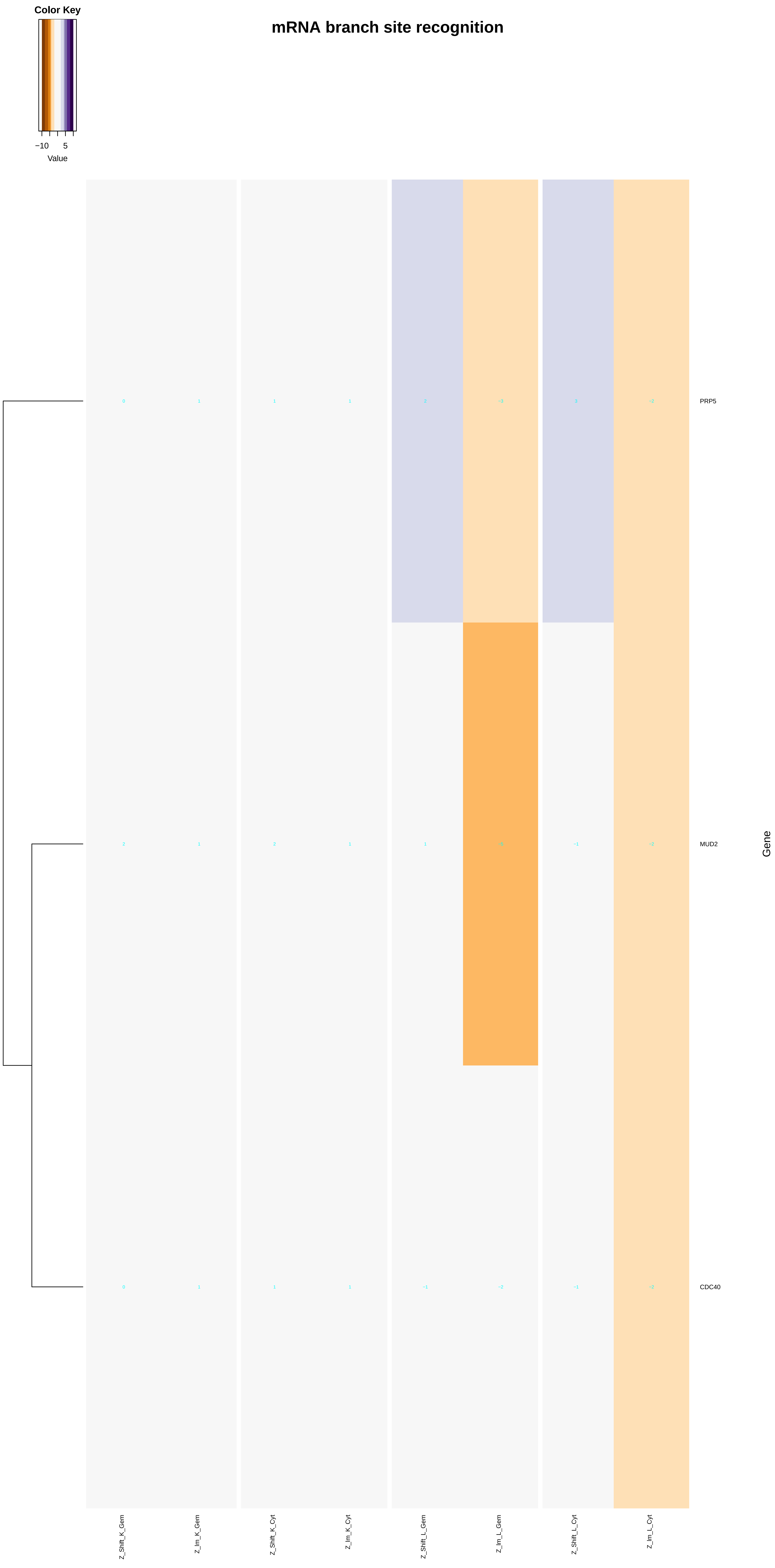
Color Key
−10
5
Value
mRNA branch site recognition
PRP5
MUD2
CDC40
Gene
Z_Shift_K_Gem
Z_Im_K_Gem
Z_Shift_K_Cyt
Z_Im_K_Cyt
Z_Shift_L_Gem
Z_Im_L_Gem
Z_Shift_L_Cyt
Z_Im_L_Cyt

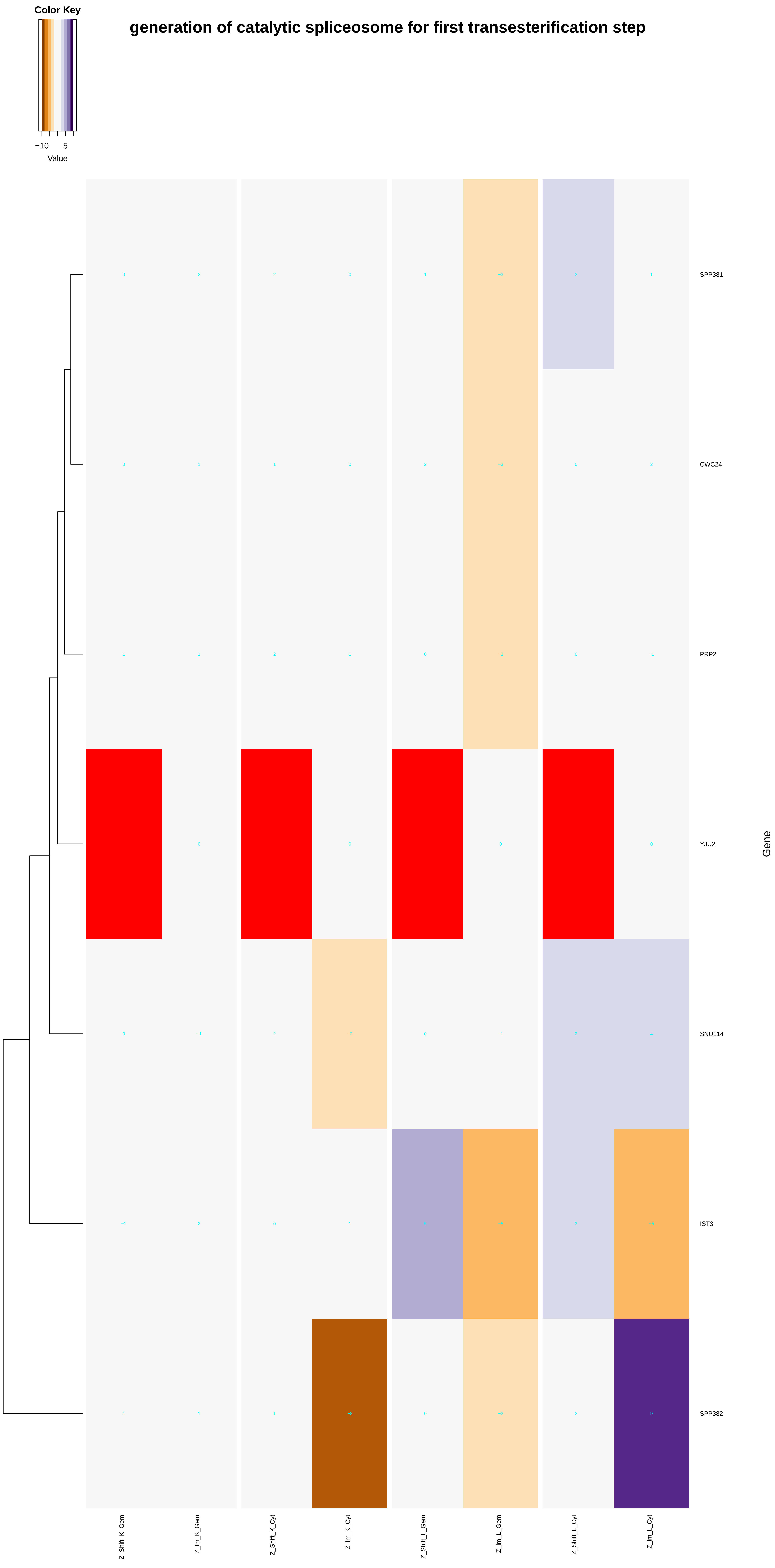

Color Key
−10
5
Value
generation of catalytic spliceosome for first transesterification step
SPP381
CWC24
PRP2
YJU2
SNU114
IST3
SPP382
Gene
Z_Shift_K_Gem
Z_Im_K_Gem
Z_Shift_K_Cyt
Z_Im_K_Cyt
Z_Shift_L_Gem
Z_Im_L_Gem
Z_Shift_L_Cyt
Z_Im_L_Cyt

generation of catalytic spliceosome for second transesterification step

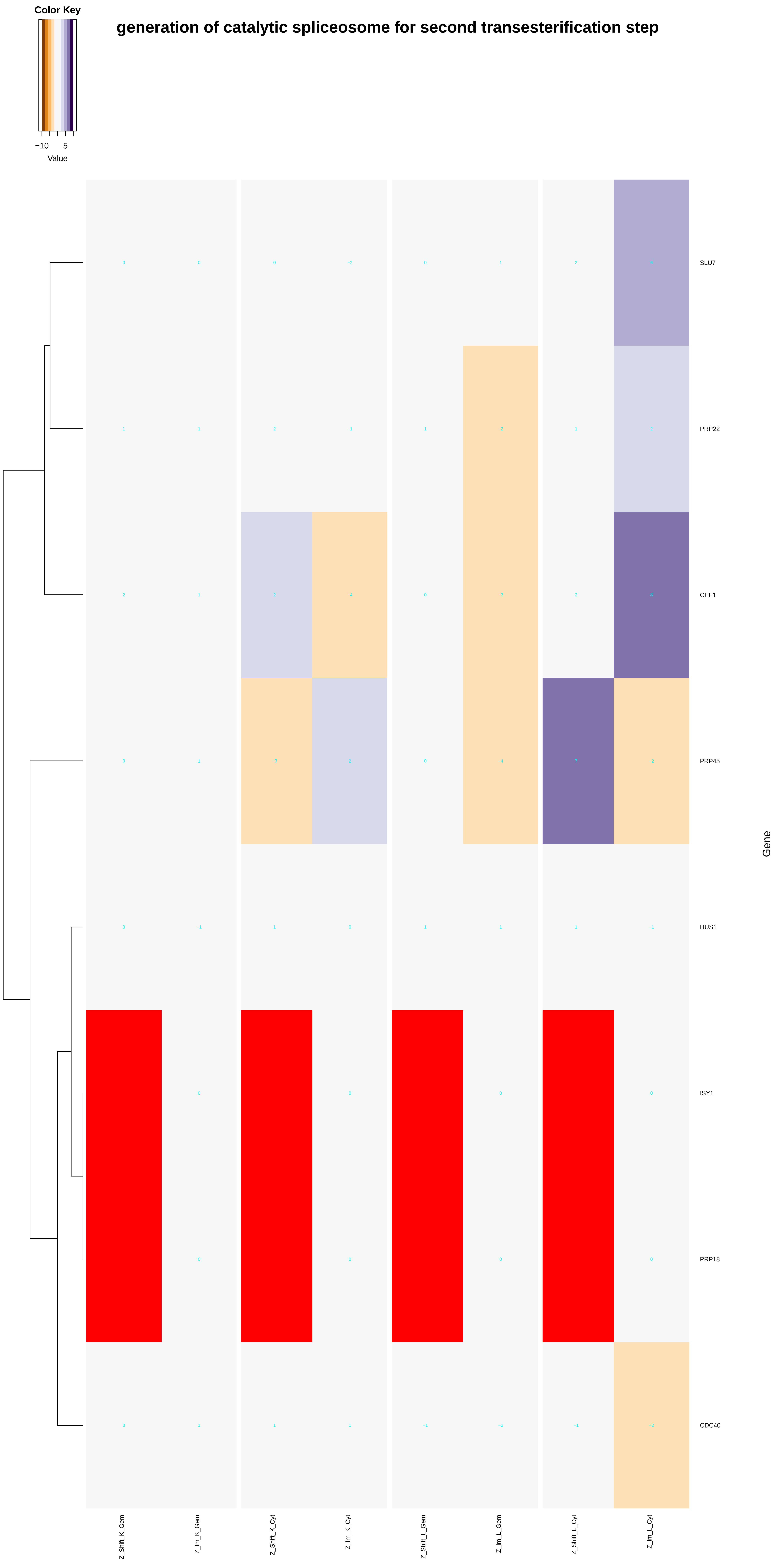

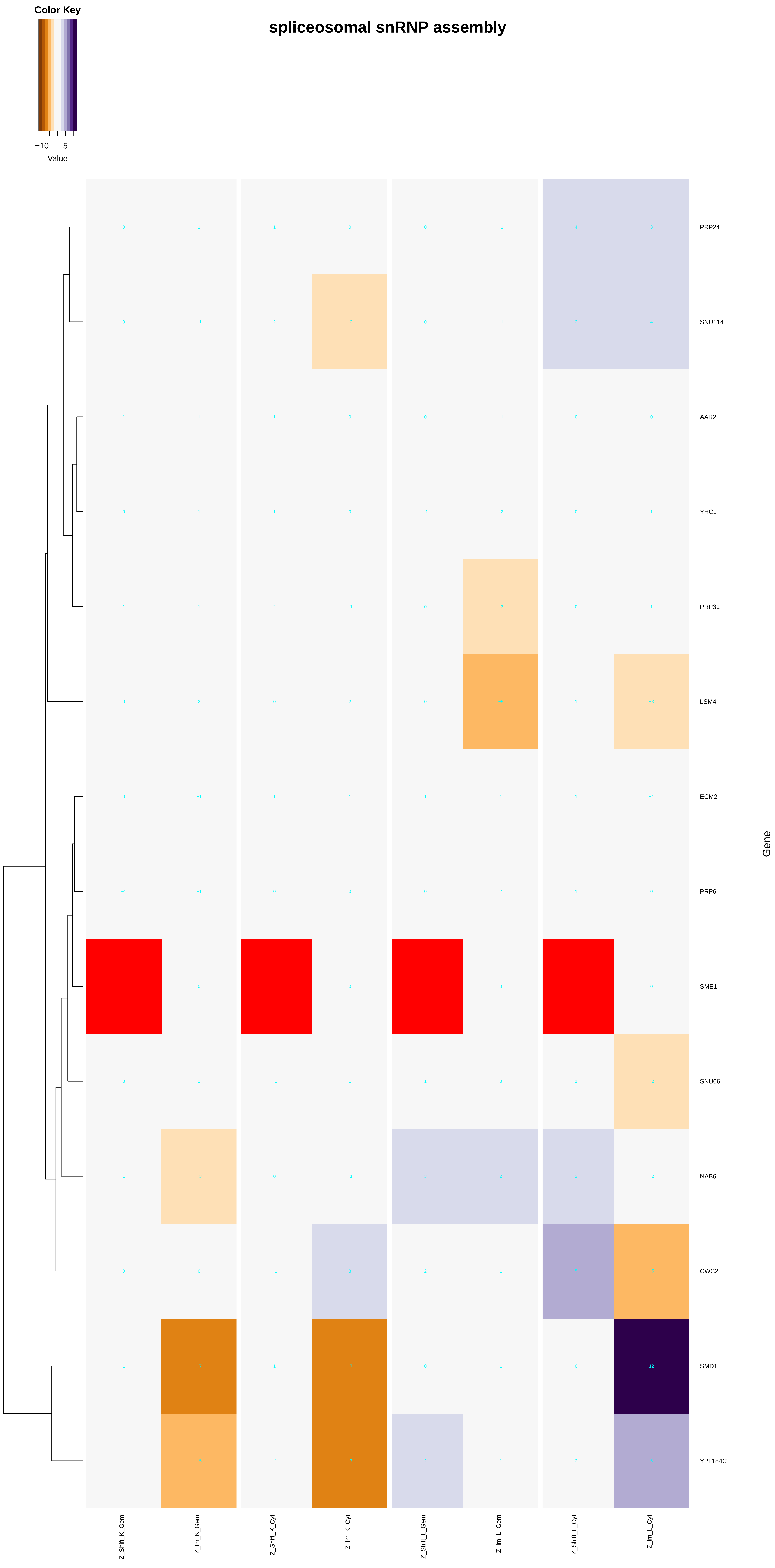

Color Key
−10
5
Value
spliceosomal snRNP assembly
PRP24
SNU114
AAR2
YHC1
PRP31
LSM4
ECM2
PRP6
SME1
SNU66
NAB6
CWC2
SMD1
YPL184C
Gene
Z_Shift_K_Gem
Z_Im_K_Gem
Z_Shift_K_Cyt
Z_Im_K_Cyt
Z_Shift_L_Gem
Z_Im_L_Gem
Z_Shift_L_Cyt
Z_Im_L_Cyt

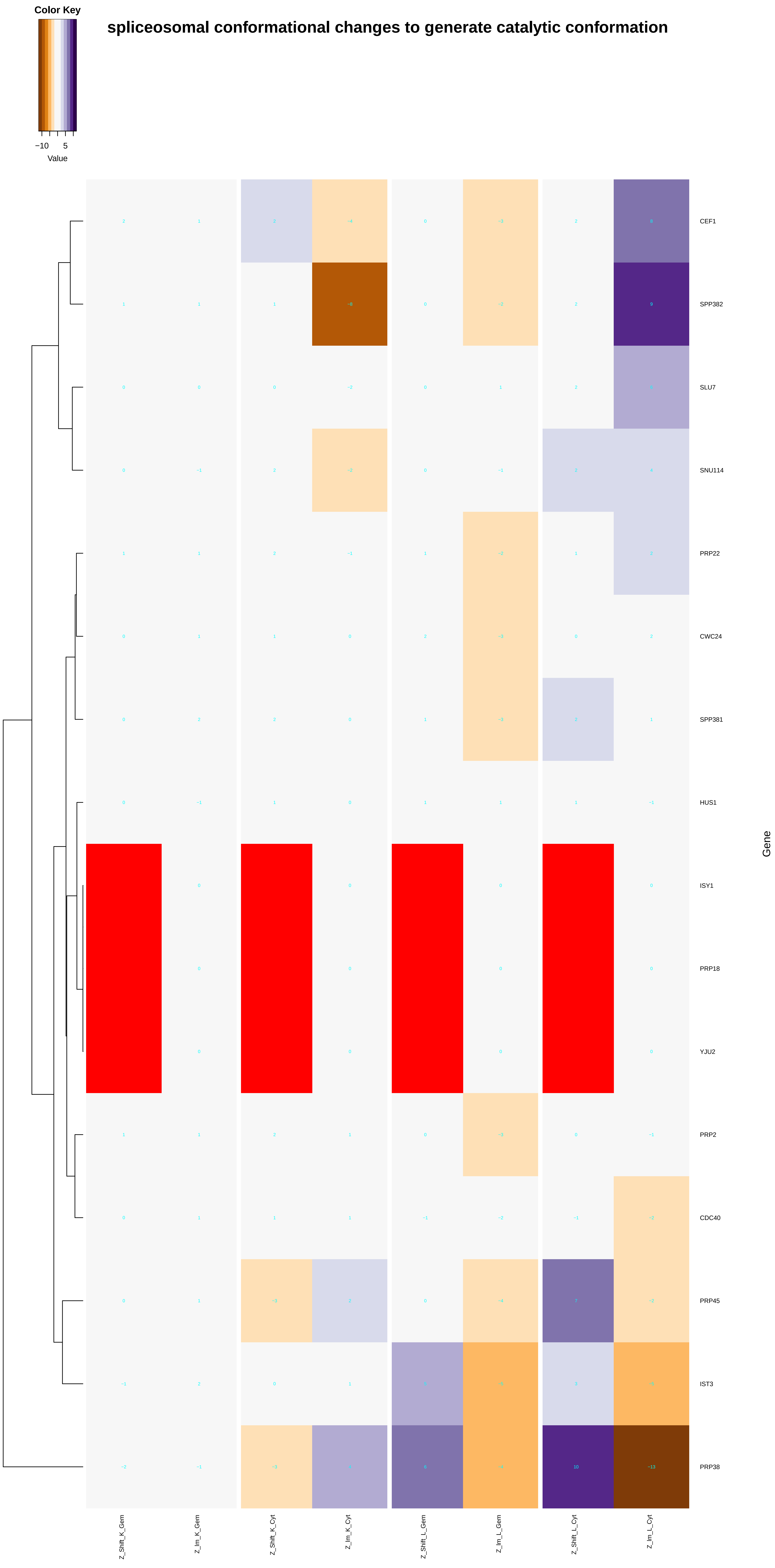
Color Key
−10
5
Value
spliceosomal conformational changes to generate catalytic conformation
CEF1
SPP382
SLU7
SNU114
PRP22
CWC24
SPP381
HUS1
ISY1
PRP18
YJU2
PRP2
CDC40
PRP45
IST3
PRP38
Gene
Z_Shift_K_Gem
Z_Im_K_Gem
Z_Shift_K_Cyt
Z_Im_K_Cyt
Z_Shift_L_Gem
Z_Im_L_Gem
Z_Shift_L_Cyt
Z_Im_L_Cyt

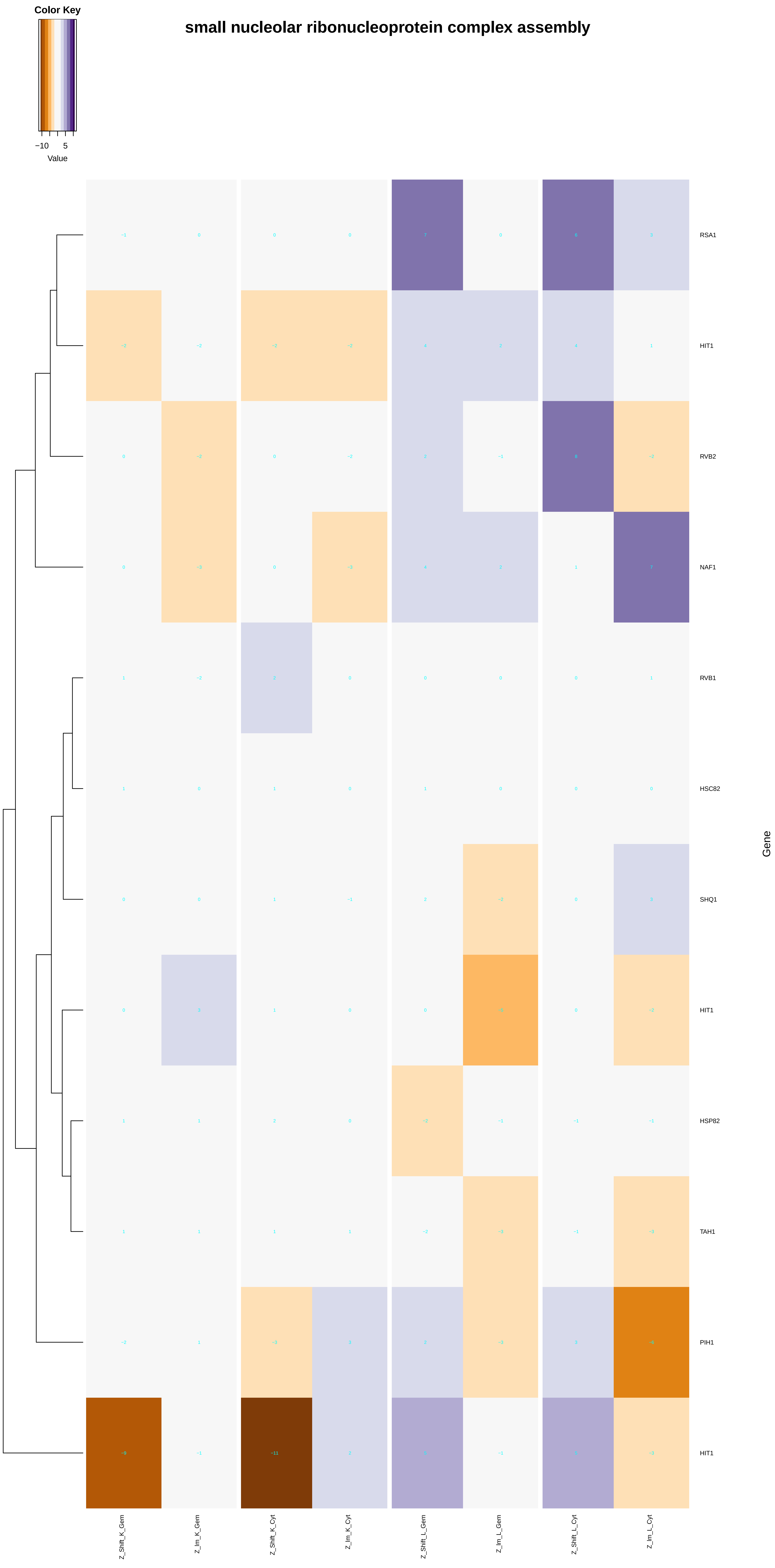
Color Key
−10
5
Value
small nucleolar ribonucleoprotein complex assembly
RSA1
HIT1
RVB2
NAF1
RVB1
HSC82
SHQ1
HIT1
HSP82
TAH1
PIH1
HIT1
Gene
Z_Shift_K_Gem
Z_Im_K_Gem
Z_Shift_K_Cyt
Z_Im_K_Cyt
Z_Shift_L_Gem
Z_Im_L_Gem
Z_Shift_L_Cyt
Z_Im_L_Cyt

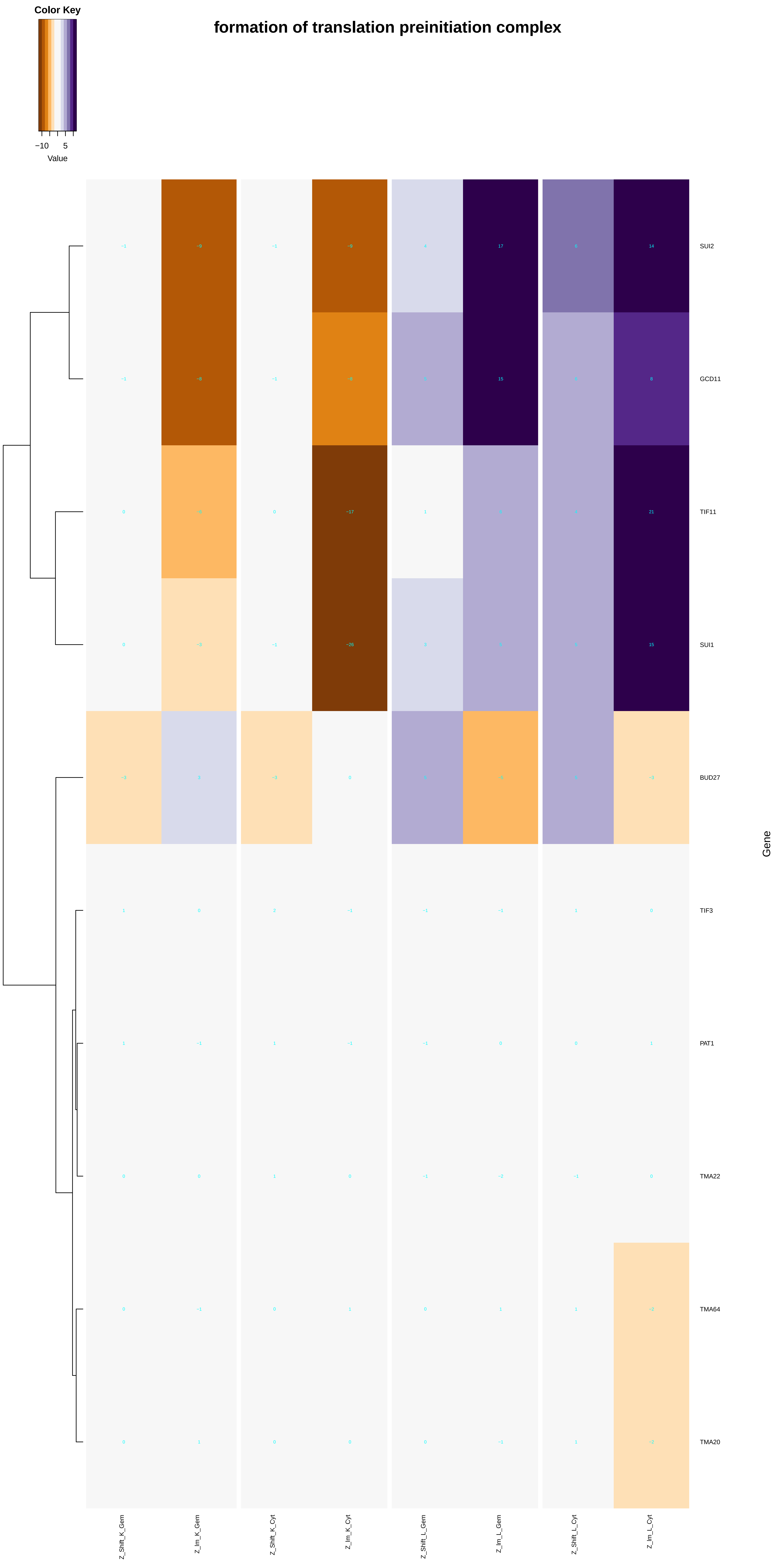

Color Key
−10
5
Value
formation of translation preinitiation complex
SUI2
GCD11
TIF11
SUI1
BUD27
TIF3
PAT1
TMA22
TMA64
TMA20
Gene
Z_Shift_K_Gem
Z_Im_K_Gem
Z_Shift_K_Cyt
Z_Im_K_Cyt
Z_Shift_L_Gem
Z_Im_L_Gem
Z_Shift_L_Cyt
Z_Im_L_Cyt

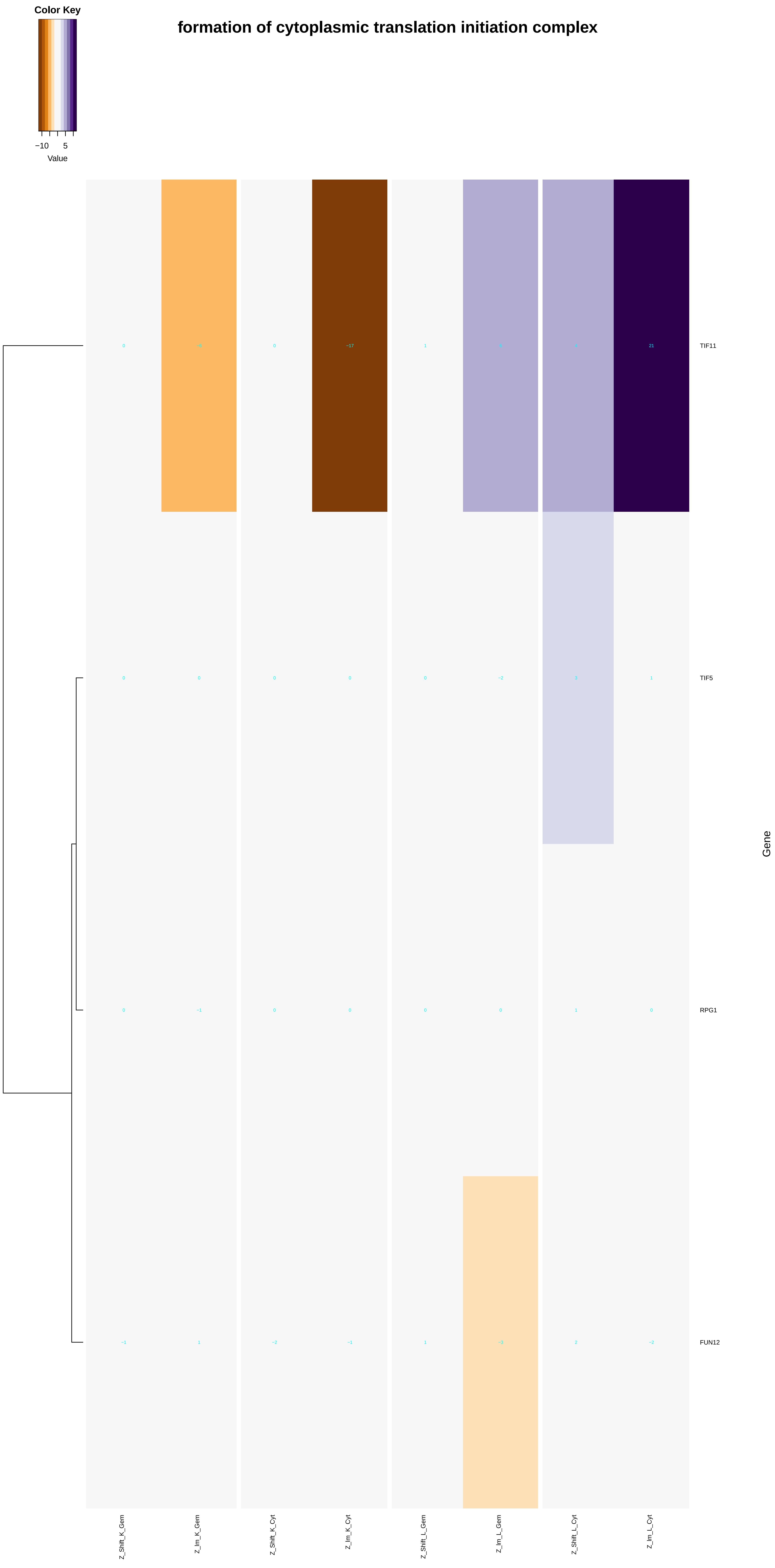

formation of cytoplasmic translation initiation complex
Color Key
−10
5
Value
TIF11
TIF5
RPG1
FUN12
Gene
Z_Shift_K_Gem
Z_Im_K_Gem
Z_Shift_K_Cyt
Z_Im_K_Cyt
Z_Shift_L_Gem
Z_Im_L_Gem
Z_Shift_L_Cyt
Z_Im_L_Cyt

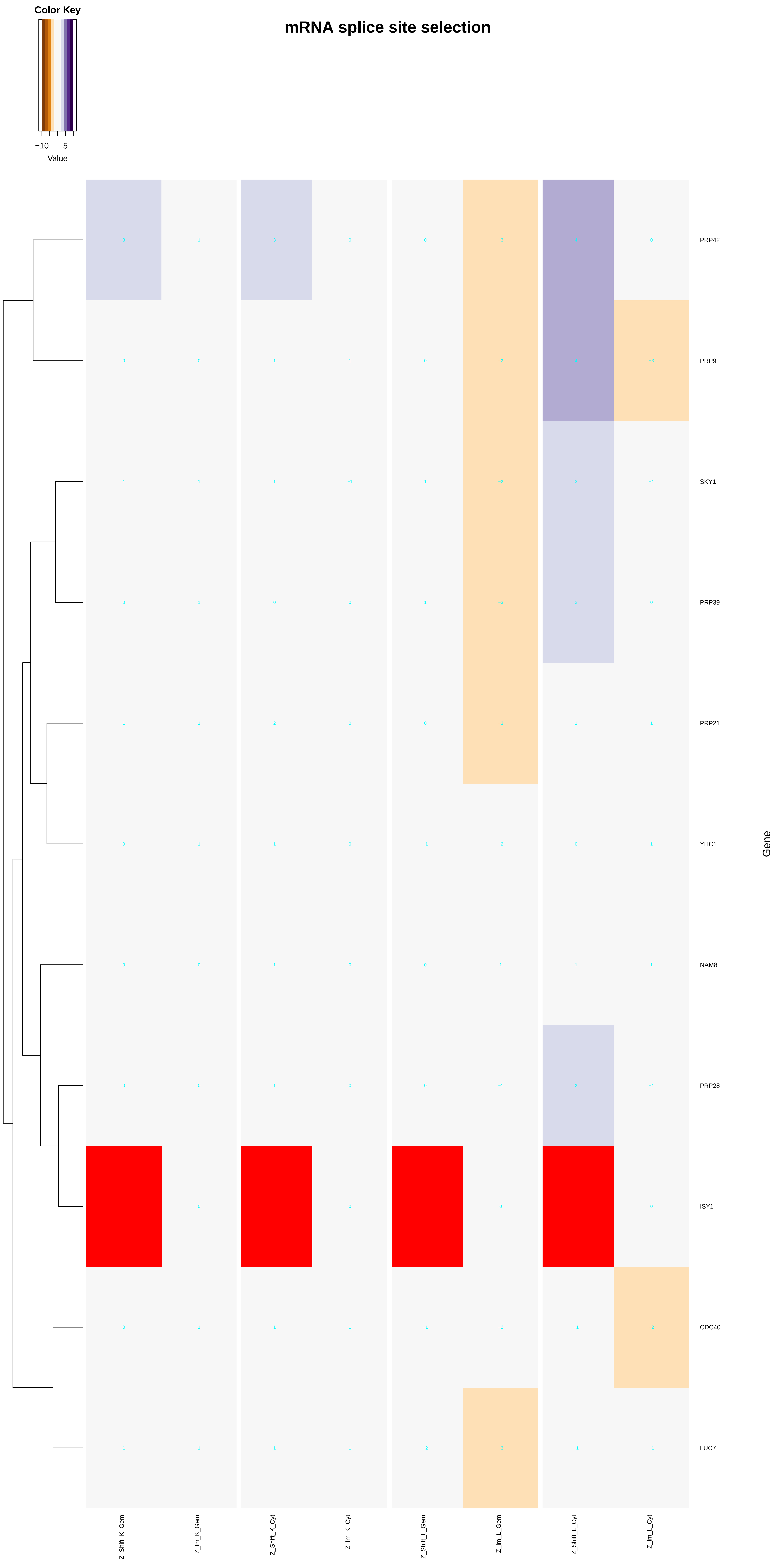
Color Key
−10
5
Value
mRNA splice site selection
PRP42
PRP9
SKY1
PRP39
PRP21
YHC1
NAM8
PRP28
ISY1
CDC40
LUC7
Gene
Z_Shift_K_Gem
Z_Im_K_Gem
Z_Shift_K_Cyt
Z_Im_K_Cyt
Z_Shift_L_Gem
Z_Im_L_Gem
Z_Shift_L_Cyt
Z_Im_L_Cyt

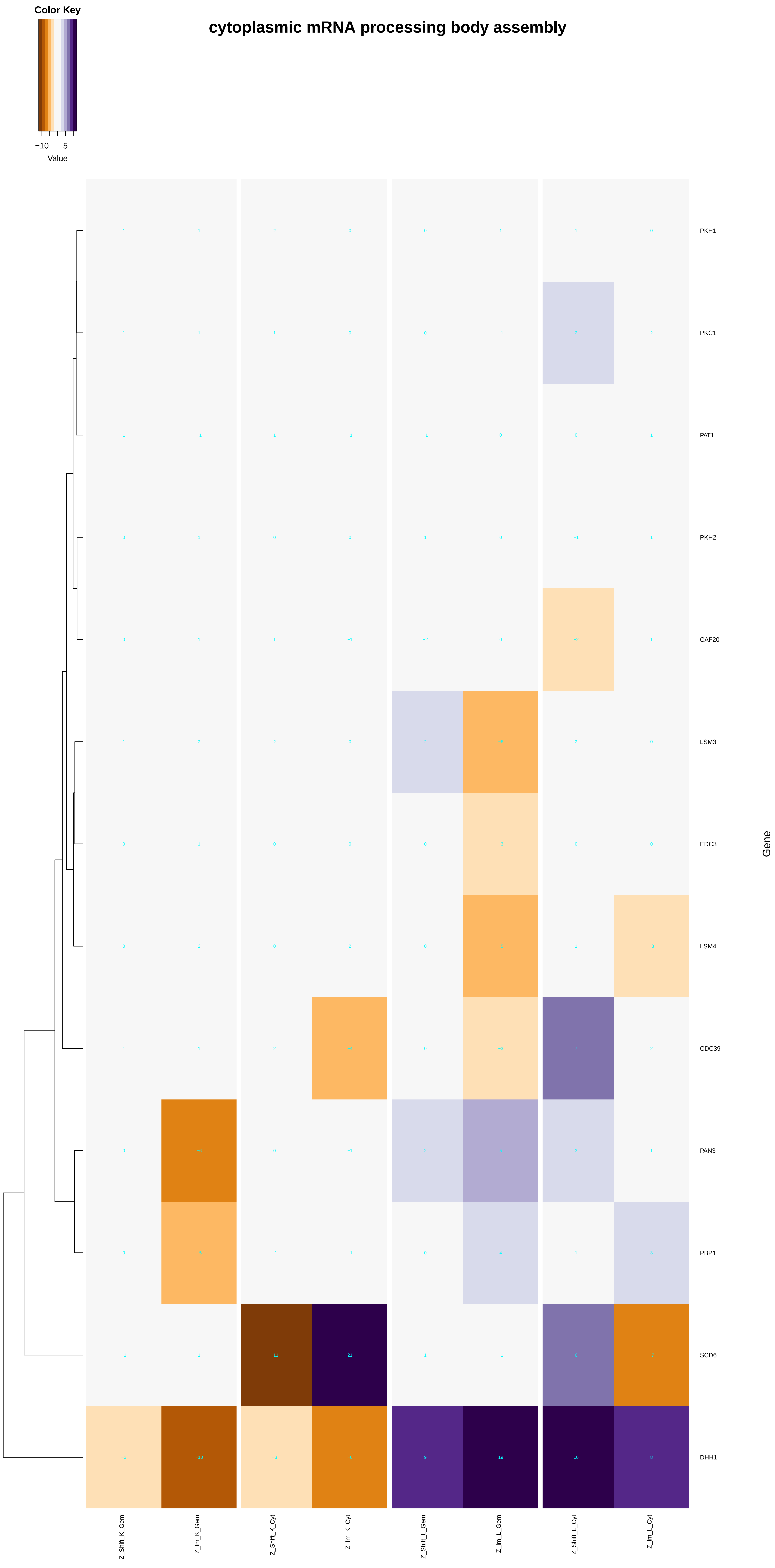
cytoplasmic mRNA processing body assembly
Color Key
−10
5
Value
PKH1
PKC1
PAT1
PKH2
CAF20
LSM3
EDC3
LSM4
CDC39
PAN3
PBP1
SCD6
DHH1
Gene
Z_Shift_K_Gem
Z_Im_K_Gem
Z_Shift_K_Cyt
Z_Im_K_Cyt
Z_Shift_L_Gem
Z_Im_L_Gem
Z_Shift_L_Cyt
Z_Im_L_Cyt

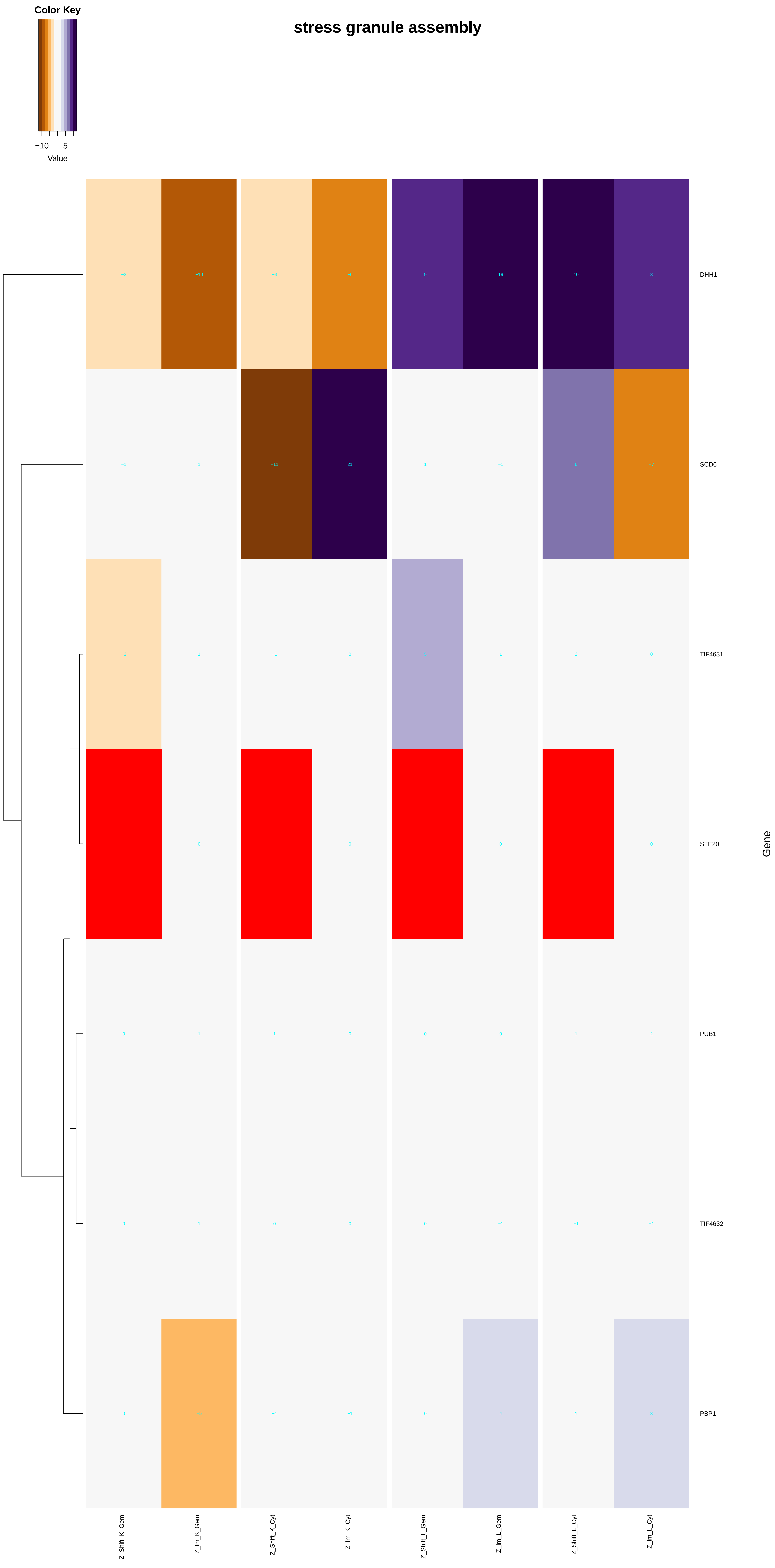
Color Key
−10
5
Value
stress granule assembly
DHH1
SCD6
TIF4631
STE20
PUB1
TIF4632
PBP1
Gene
Z_Shift_K_Gem
Z_Im_K_Gem
Z_Shift_K_Cyt
Z_Im_K_Cyt
Z_Shift_L_Gem
Z_Im_L_Gem
Z_Shift_L_Cyt
Z_Im_L_Cyt

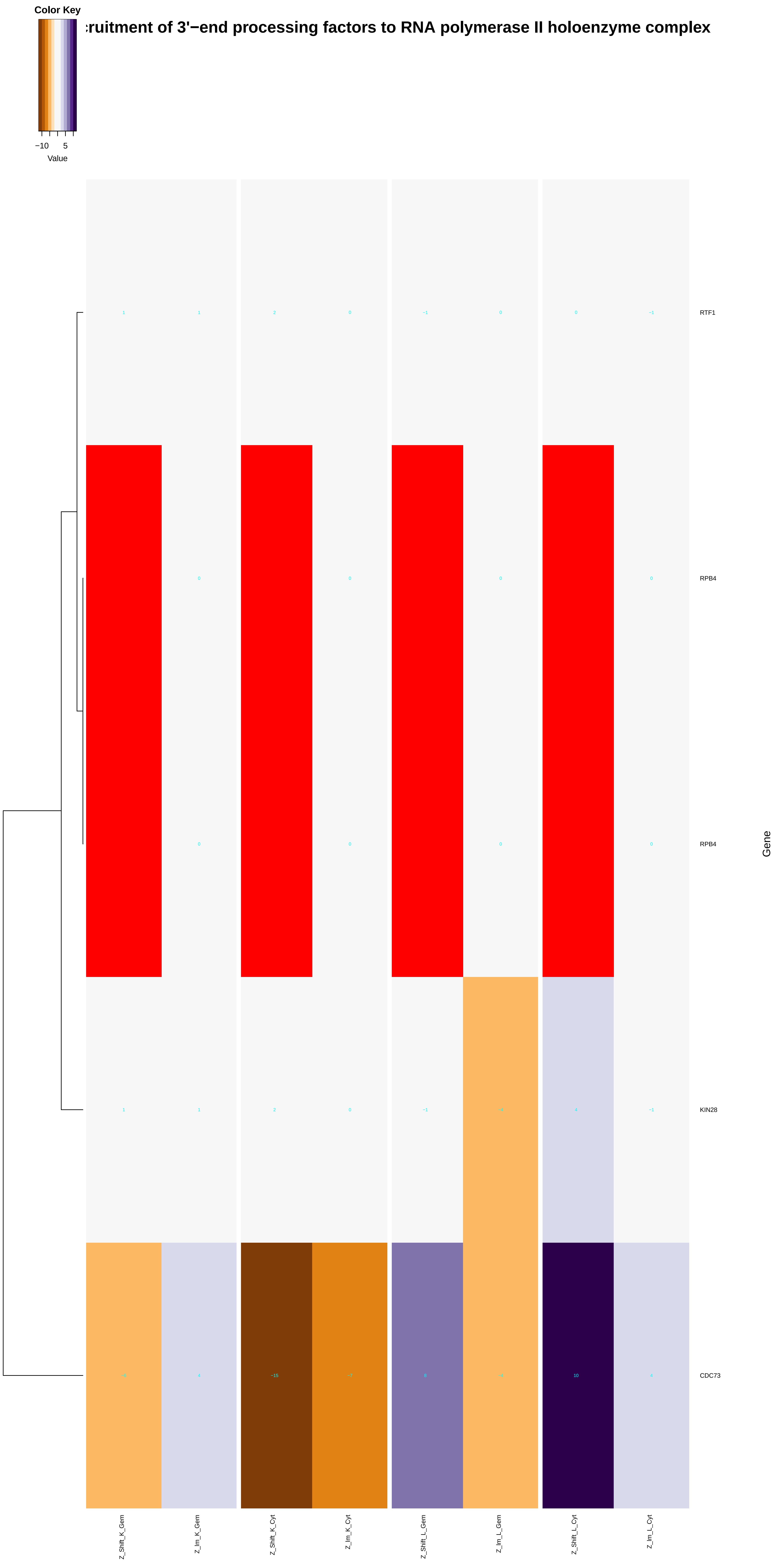
Color Key
−10
5
Value
:ruitment of 3'−end processing factors to RNA polymerase II holoenzyme complex
RTF1
RPB4
RPB4
KIN28
CDC73
Gene
Z_Shift_K_Gem
Z_Im_K_Gem
Z_Shift_K_Cyt
Z_Im_K_Cyt
Z_Shift_L_Gem
Z_Im_L_Gem
Z_Shift_L_Cyt
Z_Im_L_Cyt

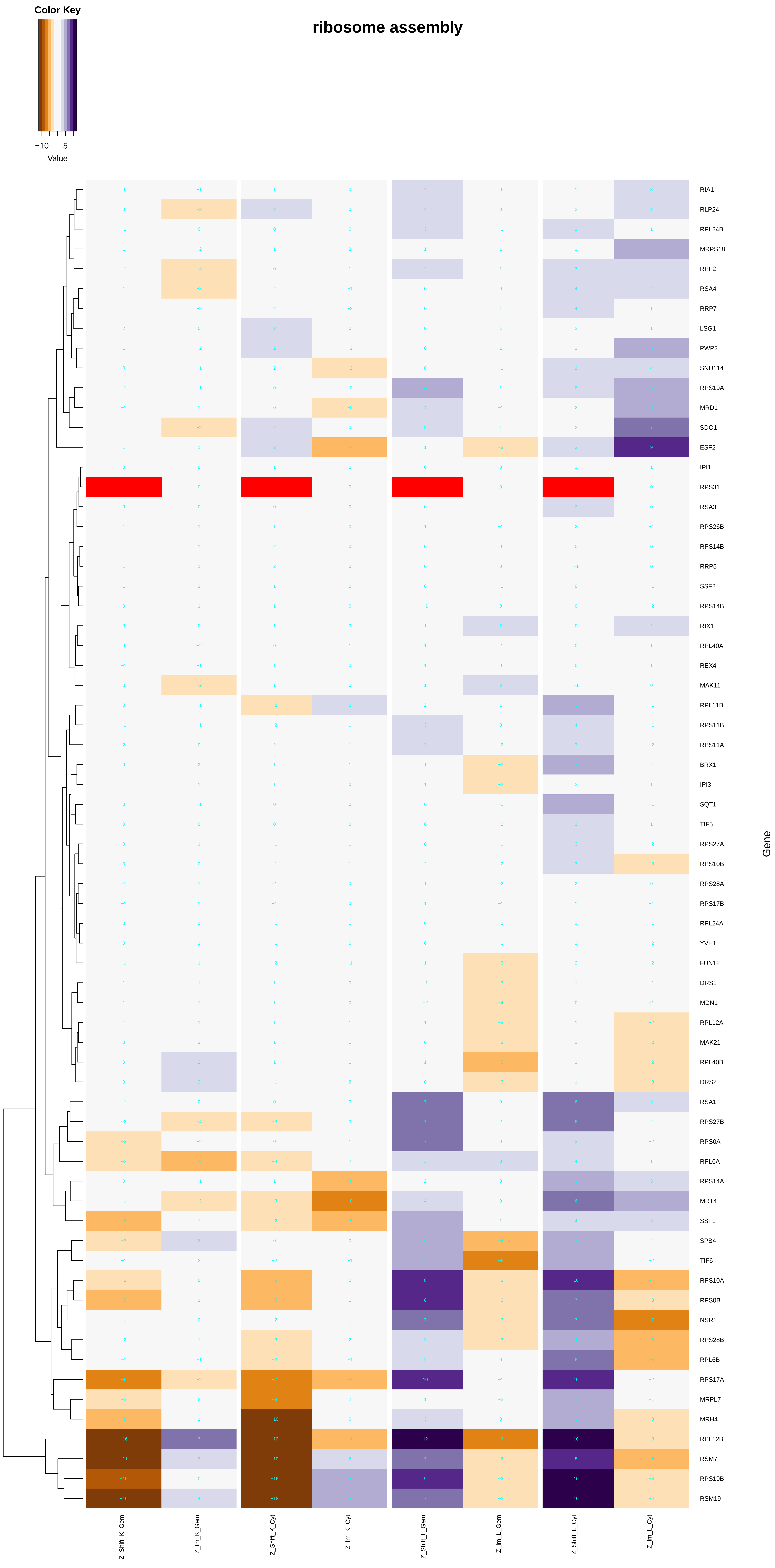

ribosome assembly
Color Key
−10
5
Value
RIA1
RLP24
RPL24B
MRPS18
RPF2
RSA4
RRP7
LSG1
PWP2
SNU114
RPS19A
MRD1
SDO1
ESF2
IPI1
RPS31
RSA3
RPS26B
RPS14B
RRP5
SSF2
RPS14B
RIX1
RPL40A
REX4
MAK11
RPL11B
RPS11B
RPS11A
BRX1
IPI3
SQT1
TIF5
RPS27A
RPS10B
RPS28A
RPS17B
RPL24A
YVH1
FUN12
DRS1
MDN1
RPL12A
MAK21
RPL40B
DRS2
RSA1
RPS27B
RPS0A
RPL6A
RPS14A
MRT4
SSF1
SPB4
TIF6
RPS10A
RPS0B
NSR1
RPS28B
RPL6B
RPS17A
MRPL7
MRH4
RPL12B
RSM7
RPS19B
RSM19
Gene
Z_Shift_K_Gem
Z_lm_K_Gem
Z_Shift_K_Cyt
Z_lm_K_Cyt
Z_Shift_L_Gem
Z_lm_L_Gem
Z_Shift_L_Cyt
Z_lm_L_Cyt

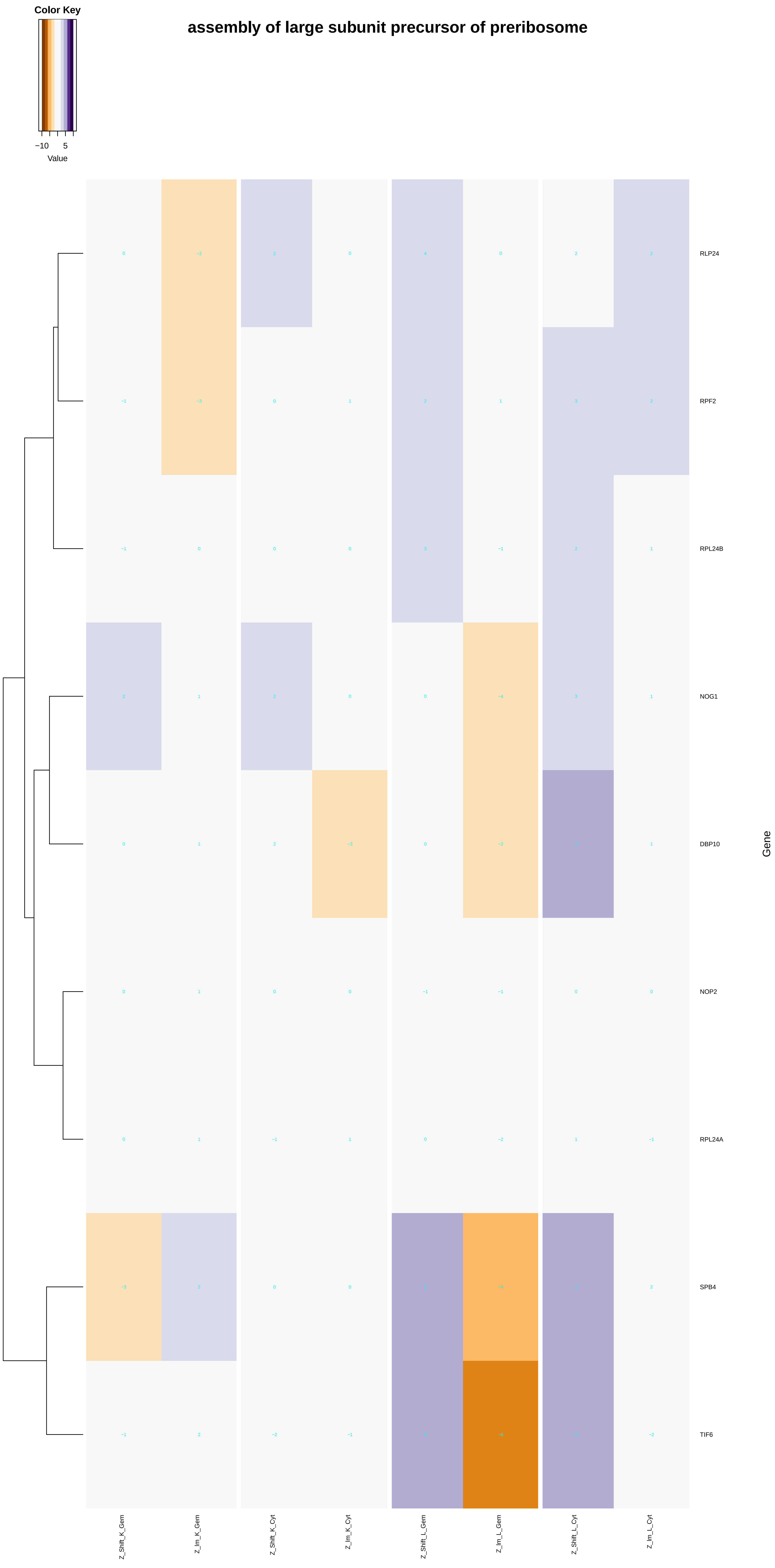

Color Key
−10
5
Value
assembly of large subunit precursor of preribosome
RLP24
RPF2
RPL24B
NOG1
DBP10
NOP2
RPL24A
SPB4
TIF6
Gene
Z_Shift_K_Gem
Z_Im_K_Gem
Z_Shift_K_Cyt
Z_Im_K_Cyt
Z_Shift_L_Gem
Z_Im_L_Gem
Z_Shift_L_Cyt
Z_Im_L_Cyt

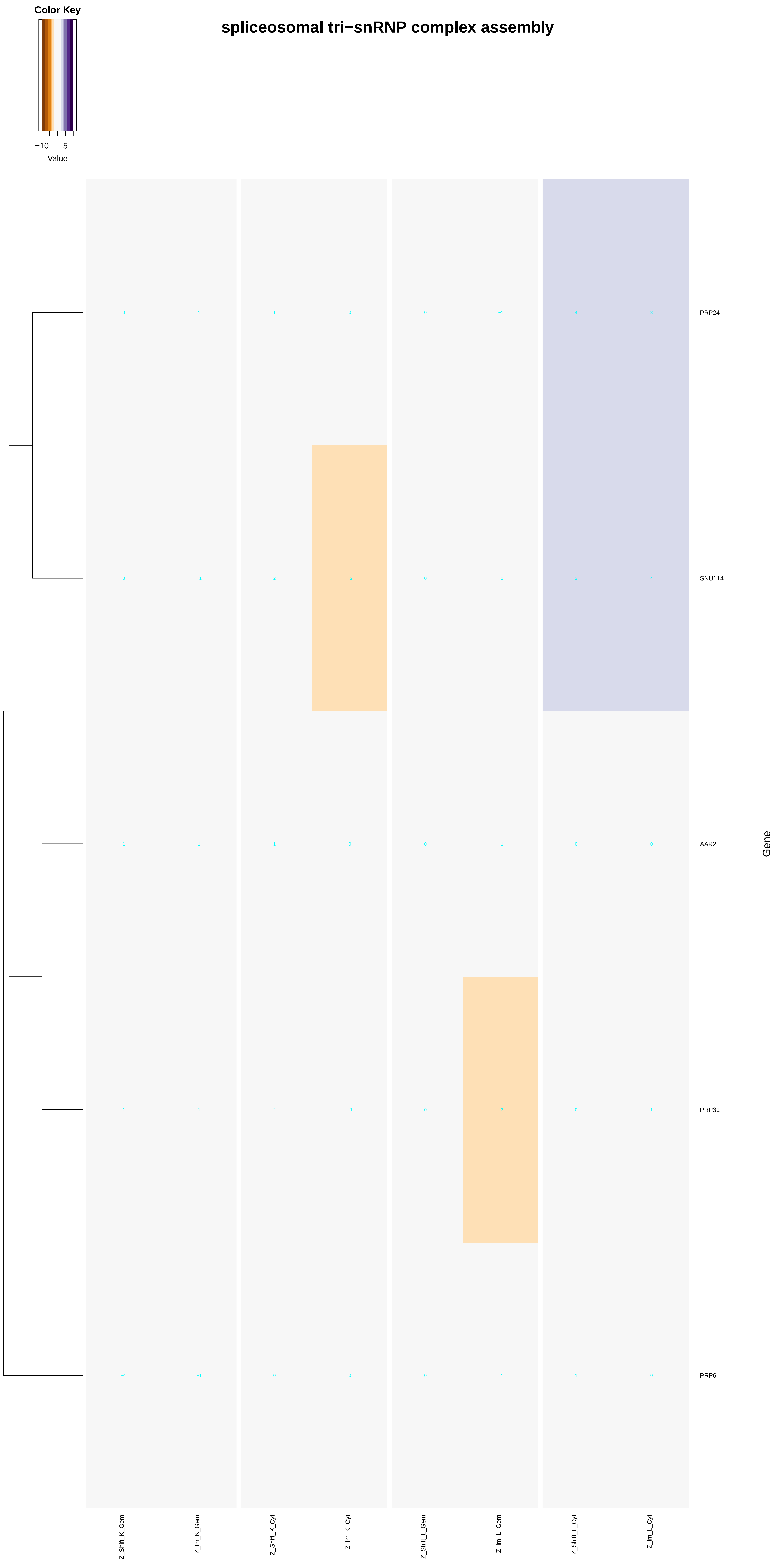

spliceosomal tri−snRNP complex assembly
Color Key
−10
5
Value
PRP24
SNU114
AAR2
PRP31
PRP6
Gene
Z_Shift_K_Gem
Z_Im_K_Gem
Z_Shift_K_Cyt
Z_Im_K_Cyt
Z_Shift_L_Gem
Z_Im_L_Gem
Z_Shift_L_Cyt
Z_Im_L_Cyt

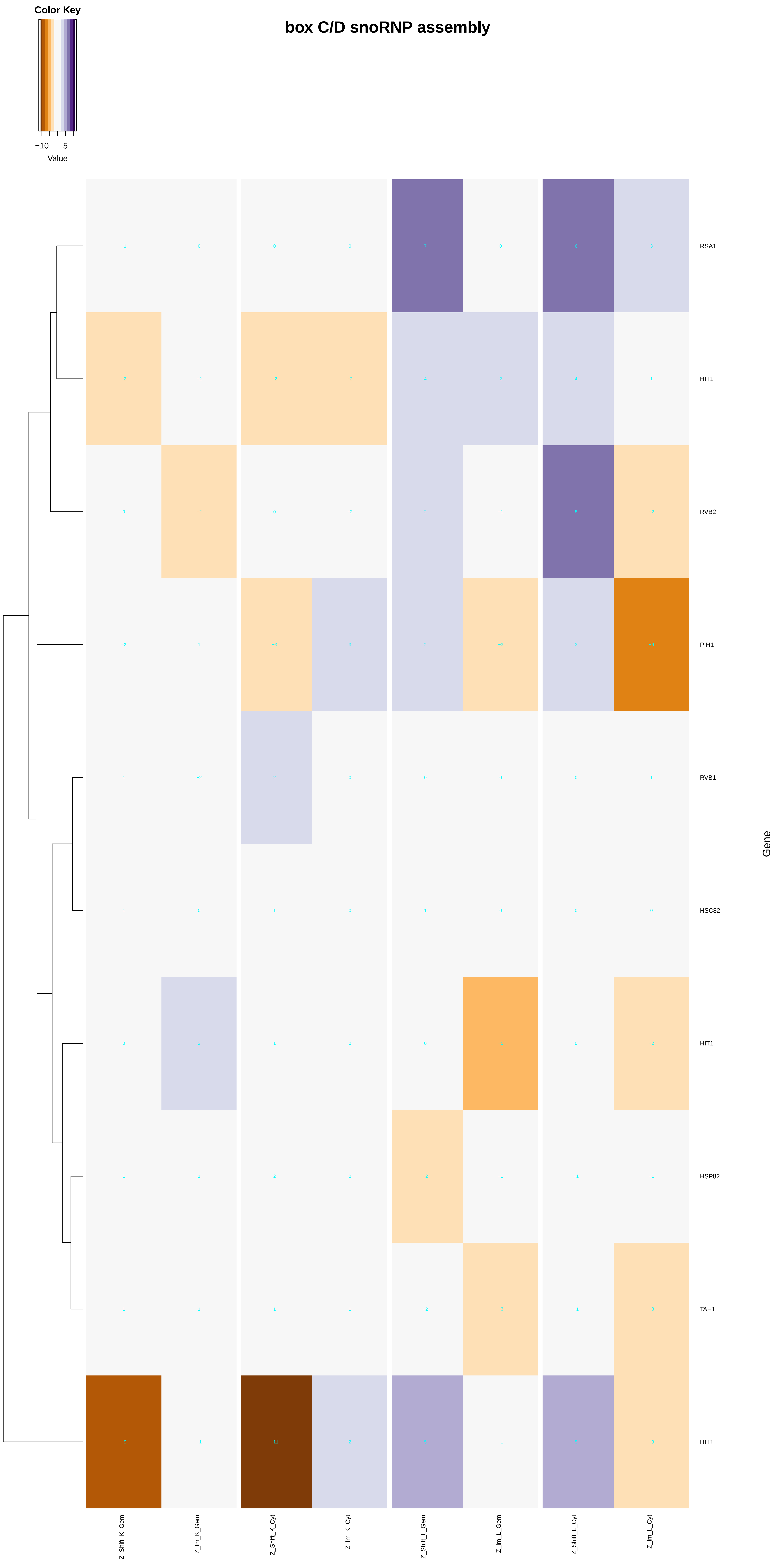
Color Key
−10
5
Value
box C/D snoRNP assembly
RSA1
HIT1
RVB2
PIH1
RVB1
HSC82
HIT1
HSP82
TAH1
HIT1
Gene
Z_Shift_K_Gem
Z_Im_K_Gem
Z_Shift_K_Cyt
Z_Im_K_Cyt
Z_Shift_L_Gem
Z_Im_L_Gem
Z_Shift_L_Cyt
Z_Im_L_Cyt

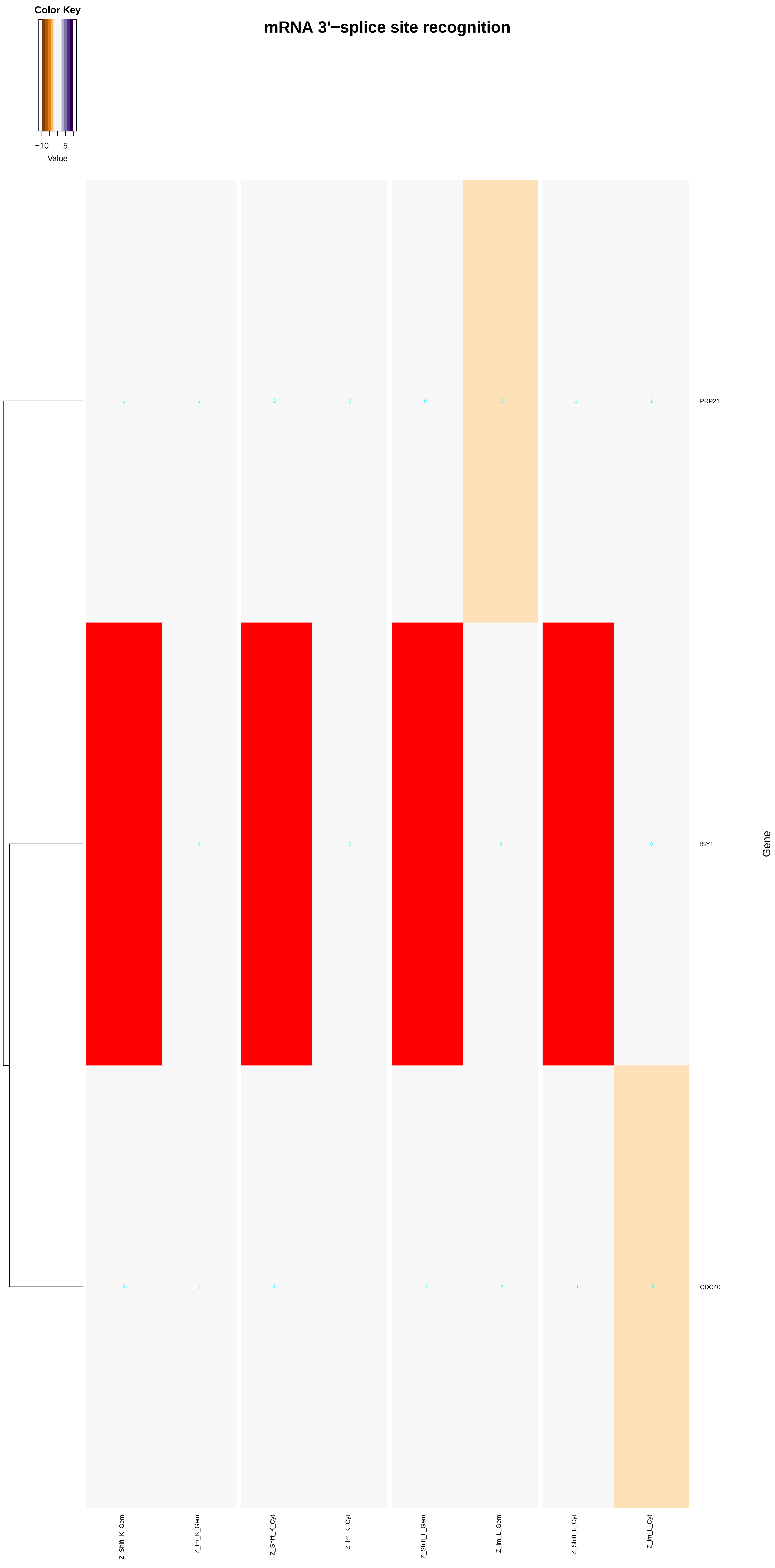

Color Key
−10
5
Value
mRNA 3'−splice site recognition
PRP21
ISY1
CDC40
Gene
Z_Shift_K_Gem
Z_Im_K_Gem
Z_Shift_K_Cyt
Z_Im_K_Cyt
Z_Shift_L_Gem
Z_Im_L_Gem
Z_Shift_L_Cyt
Z_Im_L_Cyt

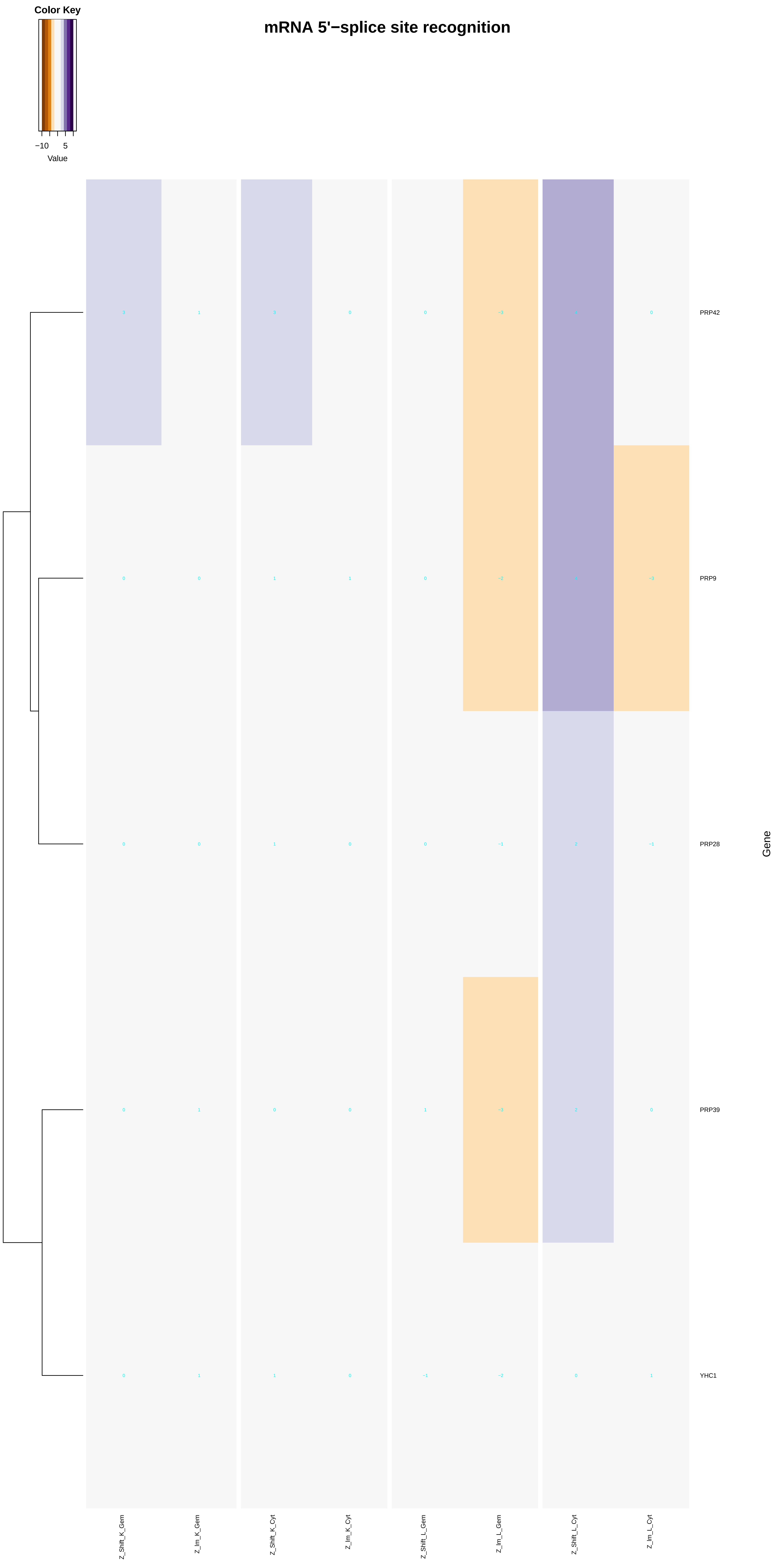
Color Key
−10
5
Value
mRNA 5'−splice site recognition
PRP42
PRP9
PRP28
PRP39
YHC1
Gene
Z_Shift_K_Gem
Z_Im_K_Gem
Z_Shift_K_Cyt
Z_Im_K_Cyt
Z_Shift_L_Gem
Z_Im_L_Gem
Z_Shift_L_Cyt
Z_Im_L_Cyt

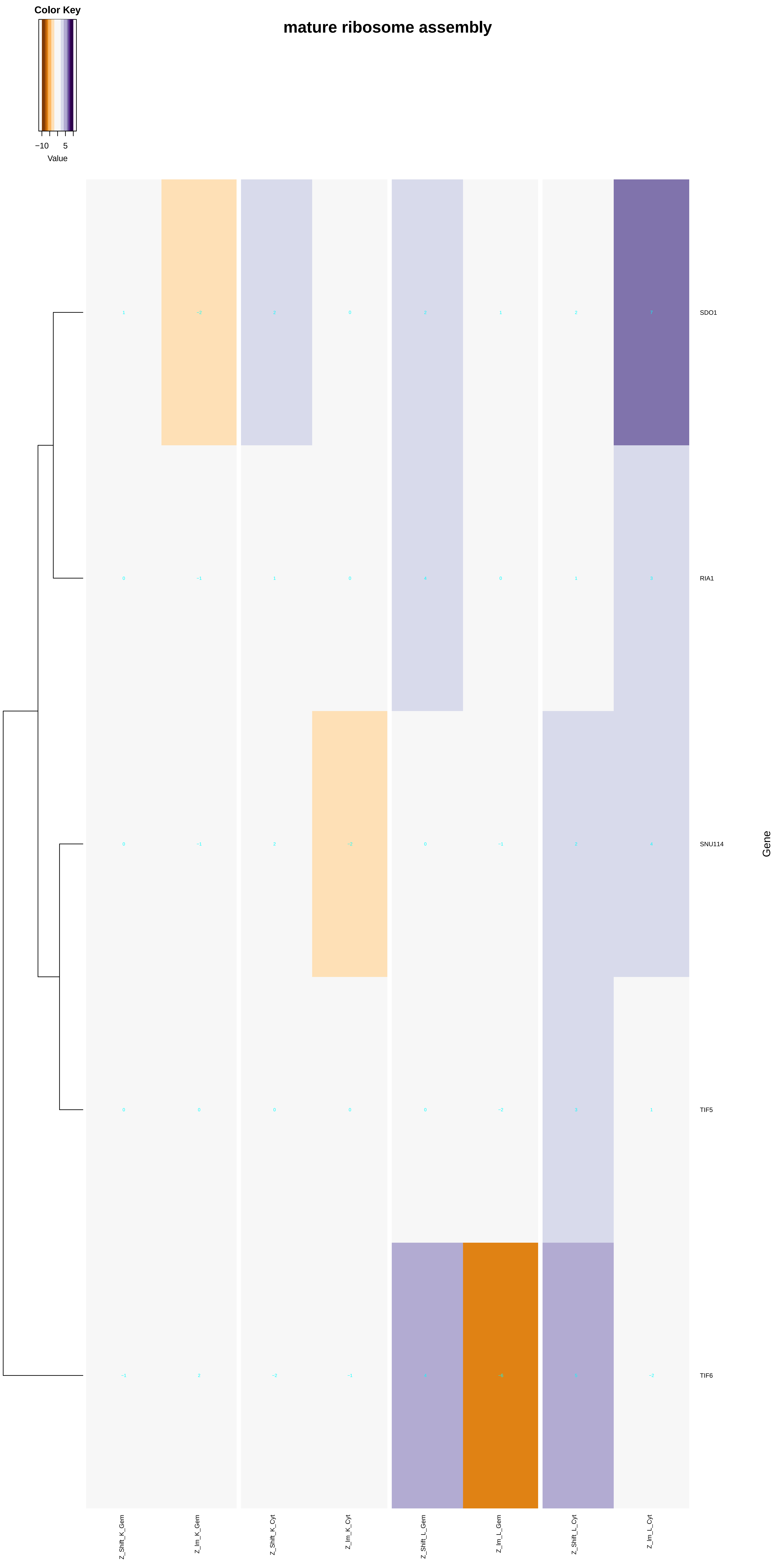

Color Key
−10
5
Value
mature ribosome assembly
SDO1
RIA1
SNU114
TIF5
TIF6
Gene
Z_Shift_K_Gem
Z_Im_K_Gem
Z_Shift_K_Cyt
Z_Im_K_Cyt
Z_Shift_L_Gem
Z_Im_L_Gem
Z_Shift_L_Cyt
Z_Im_L_Cyt
